# Special Issue: Dynamics and Nano-Organization in Plasma Membranes

**DOI:** 10.3390/membranes11110828

**Published:** 2021-10-27

**Authors:** Yenisleidy de las Mercedes Zulueta Díaz, Eva Christensen Arnspang

**Affiliations:** SDU Biotechnology, Department of Chemical Engineering, Biotechnology and Environmental Technology, University of Southern Denmark, Campusvej 55, 5230 Odense M, Denmark; ymzd@igt.sdu.dk

Cell membranes develop extraordinarily complex lipids and proteins geared to perform functions required by cells. The collective processes occurring within membranes strongly impact their cellular behavior and biochemistry; thus, understanding these processes poses unique challenges due to the often complex and multiple interactions among membrane components. The plasma membrane surface accommodates different types of lipid and protein clusters; however, the functional role of membrane surface clustering has not been fully understood yet. Super-resolution microscopy (SRM) has been extremely helpful in expanding our knowledge and changing the paradigm on both the structure and function of these clusters. These SRM techniques have been used to examine the organization and dynamics of plasma membrane components, thus giving insight into the fundamental interactions determining membrane functions.

This *Membranes* Special Issue, entitled “*Dynamics and Nano-Organization in Plasma Membranes*”, presents both an update and a comprehensive overview of recent developments in plasma membrane nano-organization. It features eight contributions, namely six research papers and two reviews. A brief descriptive summary of the scientific contributions is reported here. 

Kure et al.’s [1] review provides an in-depth overview of the performance and strengths of methodologies used in the characterization of plasma membrane nano-domains. The methods used to measure diffusion and confinement parameters are single-particle tracking (SPT), fluorescence correlation spectroscopy (FCS), image correlation spectroscopy (ICS) and fluorescence recovery after photobleaching (FRAP). Moreover, the authors reported that the line FRAP technique was applicable for the identification of the diffusion coefficient in synthetic lipid membranes [2]. The study shows, in mixtures containing cholesterol only, a correlation between the diffusion coefficient of the synthetic model membranes measured with the line FRAP method and the observed morphology. 

Kure et al. [3] also reported that k-space ICS (kICS) was a powerful image correlation spectroscopy technique and a valuable supplement to FCS for analyzing the lateral mobility of plasma membrane proteins in live cells. Using kICS, the authors studied aquaporin-9 (AQP9) diffusion in an HEK-293 cell plasma membrane and the effect of different—nanodomain perturbing—membrane and cytoskeleton-affecting drugs. It was found that the diffusion coefficient of AQP9 was 0.097 ± 0.003 µm^2^/s, which is comparable to similar studies of other aquaporins. From the kICS analysis, it can be concluded that the addition of actin polymerization-inhibiting compounds, cytochalasin D and latrunculin, did not significantly affect the diffusion coefficient of AQP9 in these cells, indicating that there was no correlation between the actin cytoskeleton network and AQP9; in addition, decreases in the diffusion coefficients were observed when adding Methyl-β-Cyclodextrin (MβCD). These observations, when combined, suggest that, unlike other AQPs (e.g., AQP4 ), AQP9 is not a nano-domain membrane protein.

In the paper by Jaszul et al. [4], the molecular dynamics of the ATP-binding cassette (ABC) transporter (ABCA1), expressed in the plasma membrane (PM) of Chinese hamster ovary cells (CHO-K1), were studied. CHOK1 cell lines stably expressing either active ABCA1 or A1 (A1G cells) or nonactive catalytic mutant ABCA1MM or MM (MMG cells) were both used in fusion with enhanced GFP (eGFP). Using spot variation fluorescence correlation spectroscopy (svFCS), ABCA1 confinement in the PM of living cells and factors influencing its molecular diffusions, such as amphotericin B (AmB) or Apolipoprotein A1 (ApoA1), were assessed. The authors showed that there was a stronger confinement in the PM domains for the active ABCA1 protein than for the nonactive catalytic mutant ABCA1MM. This molecular confinement of ABCA1 in the PM domains, of which cholesterol was not the only component, agreed with recent results, which demonstrated that AmB only affected the PM organization and diffusion parameters for nonactive ABCA1MM. Finally, ApoA1 binding influences the active ABCA1 molecular diffusion in the cell PM in relation to the cholesterol efflux process. In summary, the ABCA1 diffusion properties depend on several factors and are directly related to the PM composition and ABCA1 activity.

Sezgin et al. [5] reported a novel approach for creating supported plasma membrane bilayers (SPMBs) by bursting cell-derived giant plasma membrane vesicles using an ultrasound microfluidic device. Sezgin et al. showed that the diffusion of lipids as well as proteins in these SPMBs in the outer leaflet is preserved, suggesting that these molecules are accessible on the surface of the bilayers. The diffusivity of the tested molecules was comparable to those in the native cell membrane. The authors suggested that this model membrane system could be potentially useful for several applications in cell and membrane biology, requiring the characterization of plasma membrane dynamics.

Yang et al. [6] reviewed a recent discovery on the super-resolution nanoscopic architecture of synapses and their functional implications, with a particular focus on neuronal and immune synapses. The studies using SRM, such as structured illumination microscopy (SIM), stimulated emission depletion (STED) microscopy, single-molecule localization microscopy (SMLM) and points accumulation for imaging in nanoscale topography (PAINT), revealed unprecedented details of the nanoscopic organization and dynamics of synaptic molecules. The authors argued, by illustrating similarities in the nanoscale organization of these different types of synapses, that a common structural basis may exist for cell-to-cell communication and that researchers can find inspiration by comparing these different types of synapses. They also pointed out that emerging correlative approaches in SRM with cryogenic electron microscopy (cryo-)EM further combined their respective advantages to the point of enabling the 3D analysis of nanoclusters/structures in situ. In addition, other combined super-resolution fluorescence microscopy (SRFM) modalities, such as lattice light-sheet microscopy combined with SMLM and various 3D super-resolution microscopy techniques, could also enhance the study of native nanoscopic structures. 

In their paper, Allender et al. [7] discussed the physical origin of the plasma membrane “raft” model. The “raft theory” hypothesizes that its lipid constituents are not uniformly distributed and that sphingolipids and cholesterol float in a “sea” of unsaturated lipids. The physical properties of such heterogeneities are often attributed to a phase coexistence between two different domains. However, they are quite consistent with an alternate explanation, namely that the plasma membrane is a microemulsion of the two kinds of regions. The authors briefly reviewed a simplified theory of the plasma membrane as a microemulsion and its phase diagram. They showed how the employed model exhibited both regions of a genuine two-phase coexistence as well as a one-phase emulsion, consistent with this phase diagram. In addition, the dependence of the domain size on several physical parameters was explicated: the surface tension and bending modulus of the membrane, and the gradient energy and spontaneous curvatures of the lipids. The size of domains predicted by the theory did not vary a great deal over a variety of cell types—from 58 to 88 nm. To sum up, the authors suggested that knowledge of the dependence and independence of domains on these physical parameters could give some insight into the manner in which cells can manifest such a control over the sizes of rafts and, further, how they might manipulate their properties.

Last, Tintino et al. [8] studied the effect of menadione—also known as vitamin K3—on efflux inhibition through norA pump gene expression inhibition. The authors also studied the way menadione’s action on the cell membrane was associated with the inhibition of this pump by using transmission electron microscopy. Their results showed that the norA gene had its expression significantly diminished in the presence of this vitamin. The simulation showed that several menadione molecules were able to seep through the bilayer and allow the entry of water molecules into the hydrophobic regions of the bilayer. When present within membranes, it may cause membrane structural changes, resulting in a decline in those signaling pathways involved in norA expression. Menadione proved to be an efflux pump inhibitor with a dual mechanism; on the one hand, it affected the efflux pump by direct interaction with the norA protein, and on the other hand it indirectly inhibited norA gene expression, possibly by affecting regulators present in the membrane altered by menadione. 

In conclusion, the papers in this Special Issue provide a significant contribution to increasing our understanding of the complexity in cell membrane organization. Super-resolution microscopy techniques are highlighted as powerful tools for obtaining new information about plasma membrane nano-organization, nanodomains, lipid as well as protein diffusion, along with membrane proteins and their interaction with plasma membranes. Moreover, important contributions related to the development of new methodologies to generate model lipid membranes as well as an alternative theory were described in order to explain the physical origin of membrane domains. 

## Data Availability

Not applicable.
